# 

**Published:** 1893-06-24

**Authors:** 


### CONTENTS.

**Mr. Jonathan Hutchinson, F.R.S., on Doctors' Defences** 193

**The Royal British Nurses' Association** - - - - - 194

**Medical History from the Earliest Times.** By E. T. WITHINGTON, M.A., M.B., Oxon. LIX.—The Animists - - 195

**Contemporary Theory and Research** - - - - - - 196

**Diet in Disease.** By Mrs. ERNEST HART.—XXIV. Uric Acid as a Cause of Disease, and its Prevention by Diet - - - - - 197

**Gleanings in Science and Medicine** - - - - - - - 193

**Notes and News** - - - - - - - - - - - - 200

**The Hospital Clinic.**—BATH EYE INFIRMARY.—The Treatment of Cosmetic Defects—II. - - - - - - - - - 201

ACROMEGALE - - - - - - - - - - - - 202

NEW APPLIANCES AND THINGS MEDICAL.—Aminol—Elixir Picis Co. (Layne) - - - - - - - - - - - - 203

NEW DRUGS AND PREPARATIONS.—Chloralamide - - - - 204

**Annotations.**—Mr. Alabone and his Editorial Friends—The Brighton Life Table—A Terrible Warning to Nurses - - - 199

**The Institutional Workshop—**

WITHIN THE HOSPITALS.—The Hospitals of New York. By OUR SPECIAL COMMISSIONER - - - - - - - - - 205

FESTIVAL DINNERS AND MEETINGS.—North-Eastern Hospital for Children—The Claybury Asylum—Homes for Working Boys in London - - - - - - - - - - - - - - 206

PRACTICAL DEPARTMENTS.—Ward's Ambulance Trolly—Hospital Furniture - - - - - - - - - - - - - 207

**Editor's Letter Box.**—The Treatment of Disinfection of Scarlet Fever by Antiseptic Inunction - - - - - - - 208

**Scraps and Gleanings** - - - - - - - - - - 209

**The Book World of Medicine and Science** - - - - vii

**Medical Vacancies and Appointments** - - - - - x

### EXTRA SUPPLEMENT.—THE NURSING MIRROR.

**News from the Nursing World.**—A Royal Visit to Clerkenwell—The Royal Wedding—The Nurse Dolls—Our Holidays—"In Hospital"—Discourtesy to a Matron—Sea Breezes—An Appropriate Suggestion — Neglected Paupers — Another Queen's Nurse in Wales—What's in a Name?—American Training Schools—Short Items - - - - - - - - - cxxi

**The Royal British Nurses' Association.**—Full Text of the Charter - - - - - - - - - - - - - - cxxiii

**Surgical versus Medical Nursing** - - - - - - - cxxv

**District Nursing at Braintree** - - - - - - - cxxv

**The R.B.N.A. Charter—Where to Go** - - - - - - cxxvi

**Disinfection in Spain** - - - - - - - - - - cxxvi

**Our Examination Questions** - - - - - - - - cxxvi

**Everybody's Opinion.**—The Royal Wedding—A Paying Hospital - - - - - - - - - - - - - - cxxvii

**Hospital Discipline—Minor Appointments** - - - cxxvii

**For Reading to the Sick.**—Love - - - - - - - cxxvii

**A Sad Tragedy** - - - - - - - - - - - - - cxxvii

**A Groat's Worth of Wit—Notes and Queries** - - - cxxviii

**The Book World for Women and Nurses** - - - - - cxxix

